# Changes in Specific Substance Involvement Scores among SBIRT recipients in an HIV primary care setting

**DOI:** 10.1186/s13722-017-0101-1

**Published:** 2017-12-12

**Authors:** Carol Dawson-Rose, Jessica E. Draughon, Yvette Cuca, Roland Zepf, Emily Huang, Bruce A. Cooper, Paula J. Lum

**Affiliations:** 1 UCSF School of Nursing, 2 Koret Way, Box 0608, San Francisco, CA 94143-0608 USA; 2 Division of HIV, Infectious Diseases, and Global Medicine, UCSF Department of Medicine, San Francisco General Hospital, 1001 Potrero Ave, 307, Box 0874, San Francisco, CA 94110 USA; 3 Present Address: UC Davis Betty Irene Moore School of Nursing, 2450 48th Street, Suite 2600, Sacramento, CA 95817 USA

**Keywords:** SBIRT, Substance use, People living with HIV, Interventions

## Abstract

**Background:**

Substance use is common among people living with HIV (PLHIV) and is associated with worse outcomes along the HIV care continuum. One potentially effective clinic-based approach to addressing unhealthy substance use is screening, brief intervention, and referral to treatment (SBIRT).

**Methods:**

We conducted a two-arm randomized trial to examine the effects of a self-administered, computerized SBIRT intervention compared to a clinician-administered SBIRT intervention in an HIV primary clinic. Patients were surveyed before receiving the intervention and again at 1, 3, and 6 months. We administered the WHO Alcohol, Smoking and Substance Involvement Screening Test to determine Specific Substance Involvement Scores (SSIS) and to assign participants to categories of lower, moderate, or high risk to health and other problems for each substance. We collapsed moderate or severe risk responses into a single moderate–high risk category. Based on low rates of participation in the computerized arm, we conducted an “as treated” analysis to examine 6-month changes in mean SSIS among SBIRT intervention participants.

**Results:**

For the overall sample (n = 208), baseline mean SSIS were in the moderate risk category for alcohol, tobacco, cannabis, cocaine, amphetamine, sedatives and opioids. Of those enrolled, 134 (64.4%) received the intervention, and 109 (52.4%) completed the 6-month follow up. There was a statistically significant decline in mean SSIS for all substances except tobacco and cannabis among participants who were at moderate–high risk at baseline. We also observed a statistically significant increase in mean SSIS for all substances except amphetamines and sedatives among participants who were at lower risk at baseline.

**Conclusions:**

Substance use among patients in this urban, safety-net, HIV primary care clinic was near universal, and moderate risk substance use was common. Among participants who received the SBIRT intervention, mean SSISs decreased among those at moderate–high risk at baseline, but increased among those at lower risk at baseline over the 6-month study period. Additional research should examine the clinical significance of SSIS changes for PLHIV, which SBIRT components drive changes in substance use scores, and what other interventions might support those patients at lower risk to maintain health and engagement along the HIV care continuum.

*Trial registration* ClinicalTrials.gov study NCT01300806

## Background

In the United States, over one million people are currently living with HIV [1]. Substance use, including both alcohol and drug use, is a significant health challenge for people living with HIV (PLHIV) as well as for those most at risk for acquiring HIV. Unhealthy alcohol and drug use is one of the major drivers of HIV acquisition [2–4] and, among people already living with HIV, it contributes to low levels of engagement in HIV care [5–7], and is linked to poor medication adherence [8]. A recent review found that only 60–79% of newly diagnosed people who inject drugs in the U.S. are linked to HIV care, with 24–59% retained in care, 20–49% on treatment, and 16–42% virally suppressed [9], rates that are well below the UNAIDS 90-90-90 goal. Other studies have found that substance-using PLHIV presented later for HIV testing and care [10]; delayed linkage to HIV care and had poorer continuous engagement in HIV care [11]; and reported lower levels of being prescribed with antiretroviral therapy [12, 13]. Additional research has shown that PLHIV who use substances have lower rates of viral suppression than those who do not use substances [14], and PLHIV who inject drugs are more than twice as likely to discontinue antiretroviral therapy compared to those who do not [13]. Despite this, many PLHIV continue to use alcohol and drugs. In a national survey of adult PLHIV, 27.9% reported binge drinking and 32.5% reported illicit substance use in the prior 30 days [15].

The relationship between unhealthy substance use and poor health outcomes along the HIV care continuum underscores the critical importance of identifying PLHIV engaged in harmful use and providing evidence-based addiction treatment. The recent U.S. Surgeon General’s report on addiction calls for “integration across health care settings including primary care” [16]. A recent study showed, however, that among VA patients with alcohol use disorders, significantly fewer PLHIV received follow-up alcohol-related care compared to HIV-negative patients [17]. HIV primary care clinics may be more effective sites for screening, assessment, and intervention among those who are engaged in care [18]. In primary care, patients may present anywhere along the spectrum of substance involvement, from low risk behavior to an alcohol or substance use disorder, in recovery or during a relapse. As with other chronic illnesses, detection is an important first step, and screening can serve a dual purpose: as preventative care for those who may be at risk for problems associated with substance use, and as an opportunity for intervention for those already experiencing problems related to their substance use. Interventions may be brief counseling for those at low risk or, for those diagnosed with a substance use disorder, office-based medication-assisted treatment in the primary care setting or referral to specialty treatment by an addiction specialist. Prior studies also suggest that information technologies may be useful for improving access to behavioral interventions for substance use [19]. One strategy that has been used in various health care settings to identify harmful substance use is screening, brief intervention, and referral to treatment (SBIRT) [20].

While SBIRT has been tested in a variety of settings and populations, evidence of its efficacy as a treatment methodology for alcohol and other substance use disorders is mixed [21]. In one study conducted in four countries, SBIRT participants had lower levels of illicit substance use compared to non-SBIRT participants at follow-up, except in the United States [22]. Another meta-analysis found little evidence that SBIRT increased patients’ receipt of care to reduce alcohol consumption [23]. A more recent study found no association between a brief intervention and resolution of alcohol use disorder at follow-up in PLHIV patients of the VA [24]. A qualitative study sought to identify facilitators and barriers to implementing SBIRT in primary care, and found general patient support for SBIRT, but also identified inconsistent implementation and provider lack of time as barriers [25]. SBIRT is a potentially promising method for addressing substance use in primary care settings, and could be particularly effective in HIV primary care settings where rates of substance use are high.

Therefore, in an effort to examine SBIRT specifically in an HIV primary care setting, we developed and tested a two-arm approach to delivering SBIRT (Computer vs. Clinician). We then measured changes in self-reported substance use over 6 months, using the Alcohol, Smoking and Substance Involvement Screening Test [26].

## Methods

### Design

We conducted a two-arm randomized trial to examine the effects of a self-administered, computerized SBIRT intervention compared to a clinician-administered SBIRT intervention in an HIV clinic. The research protocol was approved by the Institutional Review Board of the University of California, San Francisco. The study methods and rationale have been described in detail elsewhere [26, 27]. Based on low rates of participation in the computer-administered arm, we conducted an “as-treated” analysis to examine the observed changes in self-reported substance use over time in participants who received either SBIRT intervention (Computer- or Clinician-administered).

### Participants

We recruited a convenience sample of patients between July 2010 and July 2011 from the waiting room of a single public hospital-based HIV clinic in San Francisco, which provides primary medical care to more than 2500 urban poor persons living with HIV/AIDS annually. Study eligibility included: (1) 18 years of age or older; (2) confirmed HIV-positive serostatus; (3) receiving HIV care at the clinic; (4) ability to provide informed consent to be a research participant and be followed over a 6-month period; and (5) ability to speak English or Spanish. All study materials were provided in both English and Spanish.

### Randomization

After baseline data collection, participants were randomized in a 1:1 ratio to receive either computer-administered or clinician-administered SBIRT. The research assistants who assessed intervention outcomes and participants’ primary care providers were blinded to study assignments.

### Intervention

The SBIRT intervention protocol consisted of three components: Screening and Assessment; Brief Intervention; and Referral to Treatment.

#### Screening and assessment

All participants underwent screening and assessment for tobacco, alcohol and other drug use with the Alcohol, Smoking and Substance Involvement Screening Test (ASSIST), which was developed by the WHO for use in primary care settings [26]. Based on a participant’s ASSIST responses, Specific Substance Involvement Scores (SSIS) were generated for each of the drug classes assessed; tobacco, alcohol, cannabis, cocaine, amphetamines, inhalants, sedatives, hallucinogens, opioids and other substances. These scores were used as the basis for the Brief Intervention portion of the SBIRT. Whether self-administered on a computer or administered by a clinician, the ASSIST could be completed in about 10 min [27].

#### Brief intervention

After screening and assessment, participants received same-day feedback in the form of a WHO ASSIST guided feedback card that detailed their substance use risk severity and received a Brief Intervention tailored to the severity of their SSIS scores and based on the principles of motivational interviewing (MI). Participants scoring at lower risk for health or other problems from their substance use received affirming, positive feedback, and safe behavior maintenance support [28]. Participants with moderate- or high-risk SSIS scores engaged in a patient-centered conversation that explored the pros and cons of continued drug use and readiness for change, and they reviewed information about specific substance use and its health complications. Action planning was offered to those participants, who were ready to make a change.

#### Referral to treatment

Participants with high-risk SSIS scores also were offered appointments to meet with the clinic social worker, who had 4 h of protected time per week to meet with participants enrolled in the study. This social worker was a skilled behaviorist with expertise as a motivational interviewing trainer and extensive knowledge and experience providing referrals for different levels of substance use treatment. Treatment options ranged from office-based addiction pharmacotherapy and counseling at the HIV clinic to medically supervised withdrawal programs, intensive outpatient treatment, and inpatient residential treatment programs in the community.

Participants randomized to the Clinician Group were to receive the screening and brief intervention procedures by a trained clinic staff member either the same day or within 1 week of study enrollment. These SBI clinicians included one Nurse and one Medical Assistant, who had more than 10 years combined experience in the HIV clinic, and who participated in two 4-h SBIRT training sessions that included how to administer and score the ASSIST, delivery of the Brief Intervention utilizing WHO ASSIST materials [29], and motivational interviewing principles and practice. Fidelity of the Clinician-administered intervention was monitored through documentation of each step in SBIRT delivery and through bi-weekly supervision meetings with senior study personnel.

Participants randomized to the Computer Group were to receive a self-administered SBIRT procedure embedded in the HIV clinic’s web-based personal health record [30]. Study staff assisted participants in setting up their electronic patient portal accounts, if they had not done so already. Participants were instructed to complete the self-administered SBIRT from a computer in the clinic or from a remote computer either the same day or within one week of study enrollment. Developed by the HIV clinic’s lead social worker and senior study staff with SBIRT and motivational interviewing expertise, the web-based SBIRT experience was designed to replicate the flow and components of SBIRT conducted by HIV clinic staff. This included interactive web-based screening and assessment using the ASSIST, motivational phrasing for delivery of the Brief Intervention (e.g. allowing the patient to select the substance to prioritize for the Brief Intervention component), and links to substance use websites and patient resources, including referrals to in-person appointments with the HIV clinic social worker—all preprogrammed into the electronic patient portal. We did not track visits to electronic resources.

### Assessment measures

Study assessments were conducted by trained research assistants. The baseline interview assessed patient demographics, including gender, sex, race/ethnicity, socioeconomic status, education, and year of HIV diagnosis, as well as frequency and severity of substance use (ASSIST). A urine specimen was collected for a urine drug screen, the results of which were not recorded in the patient’s medical record nor shared with clinic staff or providers. All measures except patient demographics were repeated at 1, 3 and 6 months.

The WHO Alcohol, Smoking and Substance Involvement Screening Test (ASSIST) is a self-report measure that consists of eight items to assess lifetime and recent non-medical substance use, including injection drug use, substance use related problems, dependency levels, and risk of current or future harm. From the ASSIST, Specific Substance Involvement Scores (SSIS) were calculated for each of the drug classes assessed; tobacco, alcohol, cannabis, cocaine, amphetamines, inhalants, sedatives, hallucinogens, opioids and other substances. The SSIS is a continuous score ranging from 0 to 31 for tobacco and 0–39 for all other substances. It is the sum of responses to items 2–7: (a) frequency of use in the past 3 months, (b) strong desire or urge to use in the past 3 months, (c) health, social, legal or financial problems due to use of a substance in the past 3 months, (d) failing to do what was normally expected of you due to use of a substance in the past 3 months, (e) anyone ever expressing concern over substance use, and (f) ever trying and failing to control, cut down or stop using. Validated cut-off points stratify scores into lower risk to health and other problems (0–10 for alcohol, 0–3 for all other substances), moderate risk to health and other problems (11–26 for alcohol, 4–26 for all other substances), or high risk (health, social, financial, legal, or relationship) consistent with a diagnosis of substance dependence (27 + for all substances).

#### Primary outcome

Our primary outcome was change in mean SSIS between baseline and 6-month follow up. Substance use risk level was defined by the mean Specific Substance Involvement Scores assessed at baseline, 1-, 3-, and 6-month study assessments. We dichotomized risk level by previously validated cut-off points [22]: lower risk (SSIS 10 or lower for alcohol, and 3 or lower for each other substance), and collapsed moderate and high risk into a moderate–high risk category (SSIS 10 or above for alcohol, and 4 or above for each other substance). Participants’ responses to the ASSIST during the SBIRT intervention procedure were not used to determine this outcome.

### Analysis

Baseline demographic characteristics and SSIS were summarized with descriptive statistics. Multilevel regression models (also called hierarchical linear models, linear mixed models, random coefficient models, and random regression models) were used to examine change over time for SSIS. Major advantages of multilevel regression over traditional repeated measures analysis include the fact that cases are not dropped due to missing observations on the dependent variable at any assessment, numeric as well as categorical predictors can be used, and methods for non-normal outcomes are available. Estimation was carried out in Stata/SE Release 14.1 [31] using maximum likelihood and the Expectation–Maximization (EM) algorithm [32–34]. Models were estimated to examine unconditional change over time (linear slope) for unit-increases over the assessment months; differences in the change trajectories as a function of baseline (initial) risk of use for each substance, and differences in the change trajectories due to the intervention by initial risk interaction. Due to strong right-skewness in many of the substance use scores (many participants’ reports of substance use were zero), estimation for the multilevel regression models was carried out with a nonparametric bootstrap with 5000 repetitions to obtain bias-corrected confidence intervals, unaffected by either extreme values or skewness [35–39].

The study sample size for the overall study was calculated based on the results of a prior clinic waiting room survey that measured current substance use in 33% of clinic patients (unpublished data). The sample size estimates were large enough to detect a 15% difference in alcohol use between the Computer and Clinician groups, with 80% power and 95% confidence.

## Results

**Fig. 1:**
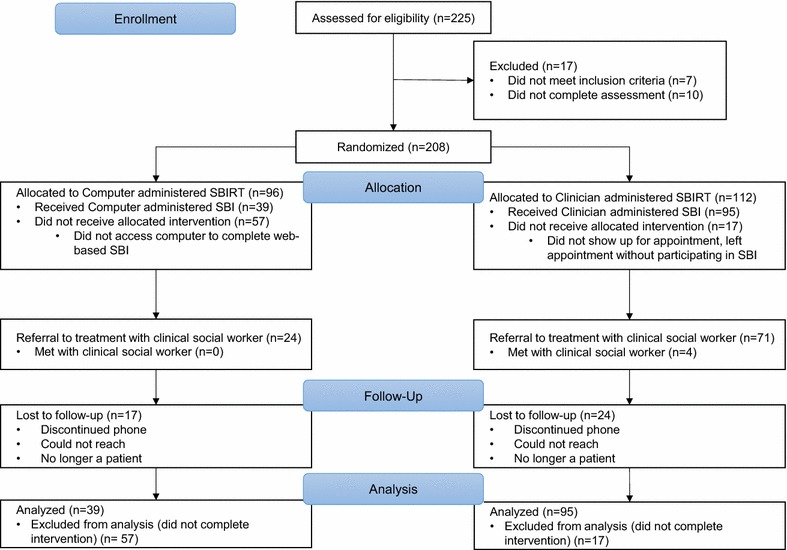
CONSORT diagram

A total of 225 people living with HIV were assessed for eligibility to participate in the study (Fig. 1). Of these, seven were excluded because they did not meet eligibility criteria, and 10 were excluded because they did not complete the baseline survey. The remaining 208 individuals were enrolled in the study and randomized. These 208 participants were primarily male (66.4%) and largely African American (39.9%), with a mean age of 45.4 years (Table 1). The majority had a high school education or less (63.4%), were unemployed (85.3%), and reported substance use (tobacco, alcohol, marijuana, stimulants, opiates, etc.) in the past 3 months (92%). The mean time since HIV diagnosis was 12.4 years. Mean Specific Substance Involvement Scores were in the moderate risk range for all substances except inhalants, hallucinogens, and other substances. SSIS were highest for tobacco, alcohol, cannabis, cocaine, and amphetamine.

**Table 1:** Characteristics of participants enrolled in an SBIRT Study (Computer and Clinician Administered) in an HIV primary care clinic (N = 208)

Variable	Total (n = 208)	Received intervention (n = 134)	No intervention (n = 74)	P
Age (mean, SD)	45.4 ± 8.5	45.0 ± 8.5	46.1 ± 8.5	0.40
Race				
African American	83 (39.9)	54 (40.3)	29 (39.2)	0.92
Caucasian	64 (30.8)	40 (29.9)	24 (32.4)	
Hispanic	35 (16.8)	24 (17.9)	11 (14.9)	
Other	26 (12.5)	16 (11.9)	10 (13.5)	
Gender				
Female	49 (23.6)	32 (23.9)	17 (23.0)	0.51*
Male	138 (66.4)	86 (64.2)	52 (70.3)	
Other	21 (10.1)	16 (11.9)	5 (6.8)	
Education (n = 202)				
High school or GED	128 (63.4)	82 (63.6)	46 (63.0)	0.94
More than high school	74 (36.6)	47 (36.4)	27 (37.0)	
Currently employed (n = 204)				
No	174 (85.3)	112 (84.9)	62 (86.1)	0.80
Yes	30 (14.7)	20 (15.2)	10 (13.9)	
Adequate income (n = 204)				
Totally inadequate	45 (22.1)	26 (20.0)	19 (25.7)	0.34*
Barely adequate	128 (62.8)	81 (62.3)	47 (63.5)	
Enough	31 (15.2)	23 (17.7)	8 (10.8)	
Health insurance (n = 205)				
No	36 (17.6)	23 (17.6)	13 (17.6)	1.00
Yes	169 (82.4)	108 (82.4)	61 (82.4)	
Clinical				
Years positive (mean, SD)	12.4 ± 7.4	12.7 ± 7.5	11.9 ± 7.1	0.44
Undetectable viral load	128 (61.5)	85 (63.4)	43 (58.1)	0.45
CD4 count (mean, SD)	512.5 ± 333.5	503.8 ± 356.6	527.8 ± 290.5	0.68
SSIS (mean, SD)^a^				
Tobacco	14.6 ± 10.8	15.1 ± 10.7	13.8 ± 11.1	0.43
Alcohol	11.2 ± 10.8	11.4 ± 11.0	10.6 ± 10.4	0.60
Cannabis	9.9 ± 10.4	10.1 ± 10.5	9.4 ± 10.4	0.65
Cocaine	9.0 ± 11.0	9.7 ± 11.4	7.6 ± 10.1	0.20
Amphetamine	8.3 ± 11.0	9.1 ± 11.5	6.9 ± 10.2	0.19
Inhalants	1.7 ± 4.9	2.0 ± 5.6	1.2 ± 2.9	0.30
Sedatives	4.3 ± 7.6	4.5 ± 8.0	3.7 ± 6.5	0.52
Hallucinogens	1.8 ± 5.3	2.1 ± 6.1	1.1 ± 3.2	0.22
Opioids	4.3 ± 8.2	4.6 ± 8.4	3.7 ± 7.8	0.50
Other	1.2 ± 4.5	1.2 ± 4.5	1.3 ± 4.5	0.88

*SSIS* Specific Substance Involvement Score, from the ASSIST measure

* Fisher’s exact

^a^Validated cut points: lower risk to health and other problems (0–10 for alcohol, 0–3 for all other substances), moderate risk to health and other problems (11–26 for alcohol, 4–26 for all other substances), high risk of severe problems (health, social, financial, legal, or relationship) consistent with a diagnosis of substance dependence (27 + for all substances)

Of the 208 individuals enrolled in the study, 134 (64%) individuals completed the baseline assessment visit and also received an SBIRT intervention. Of the 134, follow-up assessment rates were: 123 (92%) at 1-month, 106 (79%) at 3-month and 109 (81%) at 6-month; 92 (68.7%) completed all four study assessments and the intervention. Ninety-five participants with high SSIS accepted referrals to the clinic social worker, but only four met with the social worker. There were no significant baseline differences in sociodemographic characteristics or mean SSIS between those who received the intervention and those who did not (Table 1). Similarly, we found no differences between SBIRT treatment modality (Computer or Clinician) in our outcome measures of interest (SSIS) over time (data not shown).

For all substances, mean SSIS increased over time among those initially in the lower risk groups. The increase was statistically significant for all substances except amphetamines and sedatives (Tables 2, 3). However, among those individuals with moderate–high risk at baseline, mean SSIS for all substances decreased at 6 months. The decrease was statistically significant for all substances except tobacco and cannabis. For all substances, the decrease in mean SSIS for the moderate–high risk group differed significantly from the increase in mean SSIS for the lower risk group.

**Table 2:** Change in mean Single Substance Involvement Scores from baseline to 6 months among 134 patient participants who received Clinician-Administered or Computer-Administered screening, brief intervention, and referral to treatment in an HIV primary care clinic

	Change in SSIS from baseline to 6-months among those at lower risk at baseline	Change in SSIS from baseline to 6-months among those at moderate–high risk at baseline
Tobacco	+ 2.25	− 0.99
Alcohol	+ 1.79	− 7.73
Cannabis	+ 3.13	− 2.38
Cocaine	+ 1.81	− 3.10
Amphetamines	+ 0.52	− 3.65
Inhalants	+ 1.02	− 4.34
Sedatives	+ 0.52	− 8.98
Hallucinogens	+ 0.98	− 10.08
Opioids	+ 1.42	− 6.76

**Table 3:** Estimated change in Specific Substance Involvement Scores over 6 months for PLHIV SBIRT intervention participants at lower compared to moderate–high risk at baseline

Substance	Effect^a^	Coefficient^b^	95% BC CI^b, c^ Lower, upper limit
Tobacco	Months—lower	0.38	0.15, 0.65
	Months—M–H	− 0.16	− 0.44, 0.13
	Risk category	18.31	16.71, 19.90
	Month by risk	− 0.54	− 0.93, − 0.17
	Intercept^d^	1.57	
Alcohol	Months—lower	0.30	0.09, 0.51
	Months—M–H	− 1.29	− 1.84, − 0.75
	Risk category	17.44	15.44, 19.37
	Month by risk	− 1.59	− 2.19, − 1.00
	Intercept	4.06	
Cannabis	Months—lower	0.52	0.17, 1.02
	Months—M–H	− 0.40	− 0.84, 0.03
	Risk category	13.68	11.81, 15.69
	Month by risk	− 0.92	− 1.60, − 0.36
	Intercept	1.36	
Cocaine	Months—lower	0.30	0.06, 0.63
	Months—M–H	− 0.52	− 1.01, − 0.01
	Risk category	16.48	14.06, 19.03
	Month by risk	− 0.82	− 1.39, − 0.25
	Intercept	1.28	
Amphetamine	Months—lower	0.09	− 0.03, 0.21
	Months—M–H	− 0.61	− 1.22, − 0.02
	Risk category	15.95	13.56, 18.43
	Month by risk	− 0.69	− 1.32, − 0.10
	Intercept	0.75	
Inhalants	Months—lower	0.17	0.06, 0.32
	Months—M–H	− 0.72	− 1.40, − 0.09
	Risk category	7.23	4.65, 10.16
	Month by risk	− 0.89	− 1.58, − 0.24
	Intercept	0.33	
Sedatives	Months—lower	0.09	− 0.01, 0.21
	Months—M–H	− 1.50	− 2.12, − 0.83
	Risk category	12.19	9.77, 14.96
	Month by risk	− 1.58	− 2.21, − 0.92
	Intercept	0.62	
Hallucinogens	Months—lower	0.16	0.05, 0.30
	Months—M–H	− 1.68	− 3.05, − 0.02
	Risk category	11.49	7.61, 15.86
	Month by risk	− 1.84	− 3.22, − 0.18
	Intercept	0.31	
Opioids	Months—lower	0.19	0.05, 0.35
	Months—M–H	− 1.13	− 1.93, − 0.17
	Risk category	13.68	10.87, 16.53
	Month by risk	− 1.31	− 2.13, − 0.36
	Intercept	0.72	

^a^Effects in each multilevel regression model. Months—lower is simple slope over time for lower risk category; months—M–H is simple slope over time for moderate–high risk category; reference group for risk category at baseline = 0 (lower risk); month by risk is the cross-level interaction (equivalent to the difference between the two simple slopes, with lower risk as the reference category; the display with only two decimals sometimes results in rounding errors in the table)

^b^The nonparametric, bootstrapped 95% bias-corrected confidence interval from 5000 repetitions

^c^The coefficient is significant if zero is not in the interval

^d^The test of the intercept against zero is not a hypothesis test of interest

## Discussion

We conducted a screening, brief intervention, and referral to treatment (SBIRT) intervention in an urban safety-net HIV primary care clinic and detected a high prevalence of self-reported alcohol, tobacco, cannabis, cocaine, amphetamine, sedatives, and opioid use at enrollment. For all substances, the mean SSIS score for participants whose baseline substance use risk was moderate–high and who received the SBIRT intervention declined over the 6 months following the intervention, and this decrease was significant when compared to those at baseline lower risk.

While active substance use was not one of the inclusion criteria, 92% of study participants reported any substance use (tobacco, alcohol, marijuana, stimulants, opiates, etc.) in the prior 3 months. This finding is consistent with the known higher prevalence of substance use for PLHIV compared to the general U.S. national population [40]. Our results also show that, in an HIV primary care population, while mean SSISs were in the moderate range for most substances, a number of individuals were in the high risk range, as indicated by the large standard deviations for each substance. These indicators of the severity of self-reported substance use, underscore the opportunity for detection and intervention in HIV primary care settings.

We measured a significant reduction over time in the mean SSIS for alcohol − 1.59 (95% CI − 2.19, − 1.00) among participants who scored in the medium high risk categories. Several other studies that measured self-report of substance use before and following SBIRT implementation in clinical settings have been conducted and allow for a comparison with the findings of our analysis. One of the first studies to determine the effect of SBIRT in diverse clinic populations found SBI to be associated with a decrease in self-reported alcohol use at follow-up [20]. Other studies evaluating measures of alcohol use severity before and after participating in SBIRT show similar results [41]. A more recent study among PLHIV, however, found that although alcohol use declined over time, the decline was not associated with receipt of a brief intervention [24].

We also measured moderate but statistically significant decreases in mean SSISs for illicit drugs, including reductions in cocaine − 0.82 (95% CI − 1.39, − 0.25), amphetamines − 0.69 (95% CI − 1.32, − 0.10), sedatives − 1.58 (95% CI − 2.21, − 0.92) and opioids − 1.31 (95% CI − 2.13, − 0.36). Other studies have shown mixed results of the impact of SBIRT on illicit drug use following participation in SBIRT. The ASPIRE study (Assessing Screening Plus Brief Interventions Resulting Efficacy to Stop Drug Use), a 3-group randomized controlled trial for unhealthy drug use among adults from an urban primary care setting, did not demonstrate a decrease in unhealthy drug use following receipt of a primary care based SBIRT intervention [42]. Other studies have shown similar negative results of the effects of SBIRT on illicit substance use [43]. In contrast, Humeniuk and colleagues found significantly reduced SSISs among participants receiving a brief intervention compared to control participants, for all substances except opioids [22]. And Bernstein and colleagues found reductions in cocaine and heroin use among individuals receiving SBIRT [44].

In our study, we saw a reduction in mean SSIS for tobacco use among participants at moderate–high risk at baseline. Cropsey and colleagues also found that PLHIV who smoked at least five cigarettes per day significantly reduced their smoking over time following an SBIRT intervention that included a counseling session, nicotine replacement therapy, and follow-up visits, compared to those in usual care [45]. In a pilot study of 30 women living with HIV, those who received a motivational interviewing session reported significant reductions in the mean number of cigarettes smoked, compared to those who did not receive the MI intervention [46].

Surprisingly, we found that mean SSIS scores for participants who scored in the lower risk range at baseline increased over the 6 months for all substances at the same time that use dropped for those in the moderate–high risk group (this is the cross-level interaction between time and group, and it is equivalent to the difference between the simple slopes for each group). It is possible that the Brief Intervention that was given to those in the moderate–high risk groups had an important effect on reducing SSIS scores, but that the minimal intervention given to those in the lower risk group was not fully effective at keeping risk levels low. This was particularly the case for tobacco and cannabis; for both of these substances, mean SSISs in the lower risk group increased more than the scores decreased in the moderate–high risk group (the simple slope was greater in absolute value for the lower risk group only for tobacco and cannabis).

In some studies [22, 26, 42], ASSIST scores were reported as lower, moderate or high risk. Reporting and analyzing SSIS by risk categories is important because individuals who fall into the lower and moderate risk group may derive different benefits, and because brief interventions have previously been shown to be more effective among people with less severe substance use problems [20, 47]. In contrast to other studies, our outcome observation is based on a mean change score and on dichotomized risk groups, which may or may not be clinically useful distinctions. This area needs further study and exploring more effective interventions for people at lower risk for substance use related problems is an important area for future research.

While the levels of substance use self-reported among this cohort of PLHIV patients is higher than in the general U.S. national population [40], it can be difficult to make comparisons between studies because of the variability of substance use measures. For example, in a study with a safety-net primary care population, participants received an intervention based on reports of problem drug use via the Addiction Severity Index-Lite measure [43]. Because of differences between the Addiction Severity Index and the ASSIST, similar participants in each study may have been assessed at different levels of risk, and therefore may have differed in whether they qualified to receive an SBIRT intervention or not. Such differences make comparisons difficult.

Our study has several limitations. First, the data reported here were all self-reported, which could be biased due to participant recall or social desirability. Second, while these findings suggest that SBIRT delivery in HIV care settings may be associated with a decrease in the mean SSIS scores for moderate–high risk substance use, we do not have a good understanding of the clinical significance of these changes in mean scores. A decrease in the SSIS for any substance is a change in the right direction when our goal is to address substance use in HIV clinical settings. However, in the absence of a no-treatment control group it is possible that the decrease in SSIS scores across both arms of the study could be due to regression to the mean and not the intervention [48]. Third, for this analysis, which examined only those participants who received the intervention, our analytic sample may have been underpowered, despite the fact that we enrolled a sufficient number of participants into the study based on our a priori sample size calculations for a randomized trial. Notwithstanding the smaller analytic sample size, we did detect a statistically significant decrease on the moderate–high risk mean SSIS of those who received the SBIRT intervention when compared to those with lower risk scores. Fourth, while the ASSIST measure includes many drugs and does not solely capture the level of use for the substance of concern to the participant, the BI that was delivered by each modality was based on the substance of most concern to the patient. Nonetheless, our use of the ASSIST allowed us to gain a more expansive understanding of the number and types of substances used by this HIV primary care sample and this may be one benefit for using the ASSIST. Use of the ASSIST in clinical settings could have the advantage of giving providers screening and assessment information for multiple substances. Fifth, as part of our study procedures we did not adequately document the number of brief intervention visits either with a clinician or through the computer portal so we were unable to capture meaningful information about the dose of the exposure to the intervention to allow for dose–response analyses. Sixth, our findings may not be generalizable to PLHIV who are not engaged in primary care or to patients of HIV clinics that do not serve an urban safety-net population.

Further, while we collected baseline substance use and follow up data on 208 participants, and all participants were assigned to one of two SBIRT modalities, we observed a significant drop off between assessment and participation in the intervention; only 64% of participants who completed the baseline study visit actually received the intervention by either modality, leading to our decision to present an “as treated” analysis. This was particularly the case for those assigned to the computer group, which may indicate difficulties or discomfort with accessing computers and the Internet, or concern about the privacy of data entered into a computer linked to the Internet (a more detailed discussion of this phenomenon is available elsewhere [49]). In addition, very few of those referred to treatment actually met with the social worker as indicated, possibly indicating that they were not ready to take the initiative to seek out treatment for themselves, and that a more immediate and supported linkage might be needed. For participants assigned to the clinician group, this might take the form of a warm hand-off from the clinician conducting the screening to the social worker. For those using the web-based interface, a direct link in the portal to make an appointment, or some form of chat function could be useful. In addition, the 4 h per week may have occurred at a time when the participants could not participate and may have necessitated a follow-up or additional clinic visit to meet with the social worker.

## Conclusions

Unhealthy substance use erodes PLHIV’s progress at every step of the HIV continuum of care—from linkage to and retention in care, to antiretroviral adherence and viral suppression. This study suggests that among PLHIV presenting for care, HIV clinics have an opportunity to identify large numbers of patients that use multiple substances. While the study observed a significant decrease in mean self-reported risk to health and other problems among HIV clinic patients with moderate–high risk use who received a brief intervention, mean risk increased among lower risk patients. The model for offering BI or treatment in the clinic setting is important to understand, and integrated office-based addiction treatment is an important but underutilized alternative to referring patients elsewhere for specialty treatment [50, 51]. Additional research should examine whether the observed changes in ASSIST scores are clinically significant, elucidate what components of the intervention drive the reduction in moderate–high risk scores, and what other interventions might work for those at lower risk to maintain health and engagement along the HIV care continuum. This study suggests that among PLHIV presenting for care, HIV clinics have an opportunity to identify large numbers of patients that use multiple substances and to develop models for intervening.

## Abbreviations

PLHIV: people living with HIV
SBIRT: screening, brief intervention, and referral to treatment
SSIS: Specific Substance Involvement Scores
SUD: substance use disorders
AUD: alcohol use disorder
UNAIDS: Jointed United Nations Programme on HIV and AIDS
ART: antiretroviral therapy
PHP: Positive Health Program
WHO: World Health Organization
UCSF: University of California, San Francisco
VL: viral load
CI: confidence interval
ASPIRE: Assessing Screening Plus brief Intervention’s Resulting Efficacy
ED: emergency department
RCT: randomized controlled trial
